# Association between glycated hemoglobin levels and changes in aqueous humor inflammatory cytokines during sequential cataract surgeries in patients with type 2 diabetes mellitus: A retrospective study

**DOI:** 10.1097/MD.0000000000047437

**Published:** 2026-02-06

**Authors:** Jialing He, Xiaochun Li, Rui Gong

**Affiliations:** a The Department of Ophthalmology, Jinhua Hospital Affiliated to Zhejiang University School of Medicine, Jinhua, Zhejiang Province, China; b Department of Laboratory, Affiliated Jinhua Hospital, Zhejiang University School of Medicine, Jinhua, Zhejiang Province, China.

**Keywords:** aqueous humor, diabetic cataract, HbA1c, IL-6, IL-8, inflammation, surgical interval

## Abstract

Cataract is a common ocular complication of diabetes mellitus (DM), characterized by chronic inflammation and oxidative stress. Sequential bilateral cataract surgery provides a unique model to explore intraocular immune modulation under different systemic metabolic conditions. This study aimed to evaluate the relationship between preoperative glycated hemoglobin (HbA1c) levels and inter-eye variations in aqueous humor inflammatory cytokines among patients with type 2 diabetes mellitus. A retrospective analysis was performed on 88 patients with type 2 diabetes mellitus who underwent bilateral phacoemulsification cataract surgery with intraocular lens implantation between January 2023 and January 2025. Aqueous humor samples were collected at the beginning of each surgery, and the concentrations of inflammatory cytokines (interleukin-6 [IL-6], interleukin-8 [IL-8], tumor necrosis factor-alpha, monocyte chemoattractant protein-1, and interleukin-10) were measured using a multiplex immunoassay. Patients were stratified into 3 groups according to HbA1c levels (<7.0%, 7.0–8.9%, and ≥9.0%). The interocular differences in cytokine concentrations (Δcytokines) were compared among groups, and multivariate regression analysis was performed to evaluate the associations between HbA1c, surgical interval, and Δcytokines. IL-6 and IL-8 levels were significantly higher in second-eye surgeries compared with first-eye surgeries (*P* < .001). HbA1c was positively correlated with ΔIL-6 (*r* = 0.48, *P* < .001) and ΔIL-8 (*r* = 0.42, *P* = .002), independent of age, sex, and diabetic retinopathy severity. Patients with HbA1c ≥ 9.0% had nearly twofold higher ΔIL-6 than those with HbA1c < 7.0%. Longer surgical intervals (≥28 days) further amplified these inflammatory differences (interaction *P* = .031). Poor glycemic control and extended surgical intervals synergistically enhance intraocular inflammation during sequential cataract surgery in diabetic patients. Optimizing HbA1c and minimizing inter-surgery intervals may mitigate postoperative inflammatory risk and improve surgical outcomes.

## 1. Introduction

Cataract is one of the most common ocular complications of diabetes mellitus (DM), leading to visual impairment and substantially reducing patients’ quality of life worldwide. Chronic hyperglycemia promotes nonenzymatic glycation of lens proteins, oxidative stress, and disruption of the blood–aqueous barrier, thereby accelerating lens opacification and triggering intraocular inflammation.^[1,2]^ Increasing evidence suggests that the intraocular microenvironment in diabetes is characterized by elevated levels of pro-inflammatory cytokines, such as interleukin-6 (IL-6), interleukin-8 (IL-8), and tumor necrosis factor-α (TNF-α), which may contribute to exaggerated postoperative inflammation.^[3]^

Phacoemulsification combined with intraocular lens implantation remains the standard treatment for diabetic cataract. However, postoperative inflammation and cystoid macular edema occur more frequently in diabetic than in nondiabetic eyes, even after uneventful surgery.^[4]^ Sequential bilateral cataract surgery provides a valuable intraindividual model to examine ocular inflammatory responses under consistent surgical conditions. Recent studies have reported that the second-eye surgery often elicits a stronger anterior chamber inflammatory reaction, possibly due to immune sensitization or subclinical systemic activation induced by the first procedure.^[5]^ Nevertheless, the influence of systemic metabolic control on this inter-eye variation remains unclear.

Glycated hemoglobin (HbA1c), reflecting the mean blood glucose level over the preceding 8 to 12 weeks, is a reliable indicator of long-term glycemic control. Elevated HbA1c has been shown to upregulate cytokine production, increase vascular permeability, and aggravate oxidative injury, thereby amplifying ocular inflammatory responses.^[6,7]^ Hence, systemic hyperglycemia may influence local immune activation through multiple mechanisms. Sequential bilateral cataract surgery offers a unique intraindividual model to investigate ocular inflammatory responses under relatively uniform surgical conditions. Unlike previous studies that primarily compared diabetic and nondiabetic eyes or analyzed single-eye cytokine profiles, the present study focuses on inter-eye differences in aqueous humor inflammatory cytokines during sequential surgeries in patients with type 2 diabetes mellitus. By integrating systemic metabolic status, reflected by preoperative HbA1c levels, with surgical interval timing, this study aims to provide novel insights into how metabolic control and procedural factors jointly modulate intraocular inflammatory activity.

Taken together, this study aimed to investigate the changes in aqueous humor inflammatory cytokines between first-eye and second-eye cataract surgeries in patients with type 2 diabetes and to assess their relationship with preoperative HbA1c levels and surgical interval.

## 2. Materials and methods

### 2.1. Study design and participants

This study was approved by the Ethics Committee of Affiliated Jinhua Hospital, Zhejiang University School of Medicine. This retrospective observational study was conducted in the Department of Ophthalmology at Jinhua Central Hospital from January 2023 to January 2025. The study adhered to the tenets of the Declaration of Helsinki. Informed consent for the use of anonymized data and aqueous humor samples was obtained from all participants. A total of 97 patients with type 2 diabetes mellitus (T2DM) who underwent sequential bilateral cataract surgery during the study period were retrospectively screened. Of these, 88 patients fulfilled the eligibility criteria and were included in the final analysis.

Inclusion criteria were as follows:

1. diagnosis of T2DM according to the American Diabetes Association criteria;
2. age between 40 and 80 years;
3. there is bilateral senile cataract, a sequential cataract phacoemulsification combined with intraocular lens implantation surgery is required;
4. availability of preoperative glycated hemoglobin (HbA1c) measurements obtained within 3 months before the first surgery; and
5. successful collection of aqueous humor samples from both eyes.

Exclusion criteria included:

1. history of ocular trauma, uveitis, glaucoma, or prior retinal surgery;
2. active ocular infection or inflammation at the time of surgery;
3. occurrence of intraoperative or postoperative complications such as posterior capsule rupture or vitreous loss;
4. presence of systemic autoimmune or inflammatory diseases; and
5. ongoing systemic corticosteroid or immunosuppressive therapy.

### 2.2. Preoperative assessment

All patients underwent comprehensive ophthalmic evaluation including best-corrected visual acuity, slit-lamp biomicroscopy, intraocular pressure measurement, and fundus examination. Diabetic retinopathy (DR) was graded according to the International Clinical DR Severity Scale using fundus photography or optical coherence tomography. Systemic parameters including diabetes duration, fasting plasma glucose, HbA1c, and comorbidities (e.g., hypertension, nephropathy) were also recorded. Laboratory personnel performing the cytokine assays were masked to patients’ clinical characteristics, HbA1c levels, and surgical sequence to minimize measurement bias.

### 2.3. Surgical procedure

All surgeries were performed by the same experienced surgeon using a standardized phacoemulsification technique (INFINITI Vision System, Alcon). Topical anesthesia was achieved with 0.5% proparacaine hydrochloride eye drops. A 2.2-mm clear corneal incision was made, followed by continuous curvilinear capsulorhexis, hydrodissection, phacoemulsification and nucleus aspiration, cortical aspiration, and in-the-bag intraocular lens implantation. The same surgical technique, instruments, and viscoelastic materials were used for both eyes. Postoperatively, all patients received 0.5% levofloxacin and tobramycin-dexamethasone eye drops 4 times daily with gradual tapering over 4 weeks. The interval between surgeries of the 2 eyes (in days) was recorded and categorized into 3 groups: ≤14 days, 15 to 27 days, and ≥28 days. The interval between first-eye and second-eye surgeries was mainly determined by operating room scheduling and patient preference, and was not routinely adjusted based on HbA1c levels or diabetes-related systemic complications.

### 2.4. Aqueous humor sample collection and cytokine measurement

At the beginning of each surgery, before any intraocular manipulation, approximately 120 μL of aqueous humor was aspirated from the anterior chamber using a 30-gauge needle through the peripheral corneal incision. Samples were immediately transferred into sterile Eppendorf tubes, placed on ice, and stored at −80°C until analysis. Cytokine concentrations were measured using a multiplex bead-based immunoassay (Luminex 200 System, Luminex Corp., Austin) according to the manufacturer’s instructions. The panel included IL-6, IL-8, TNF-α, monocyte chemoattractant protein-1 (MCP-1), and interleukin-10 (IL-10). All samples from the same patient (both eyes) were analyzed in the same batch to minimize inter-assay variability. Measurements below the detection limit were substituted with half of the lower limit of quantification (LLOQ/2).

### 2.5. Grouping and variables

Patients were divided into 3 subgroups according to preoperative HbA1c level: group A: HbA1c < 7.0%; group B: HbA1c 7.0% to 8.9%; group C: HbA1c ≥ 9.0%. The primary outcome was the inter-eye difference in aqueous cytokine levels (Δcytokine = second-eye − first-eye). Secondary outcomes included individual cytokine concentrations, postoperative inflammation parameters, and their associations with HbA1c, DR stage, and surgical interval.

### 2.6. Statistical analysis

All statistical analyses were performed using SPSS version 27.0 (IBM Corp., Armonk) and R version 4.2. Continuous variables were expressed as mean ± standard deviation or median (interquartile range, interquartile range [IQR]) according to data distribution, and categorical variables were presented as frequency (percentage). Comparisons between first-eye and second-eye cytokine levels were conducted using paired *t* tests or Wilcoxon signed-rank tests. Differences among HbA1c subgroups were analyzed with 1-way ANOVA or Kruskal–Wallis tests, followed by Bonferroni correction for multiple comparisons. The relationships between Δcytokine values and HbA1c levels were examined using multivariable linear regression models adjusted for potential confounders, including age, sex, diabetes duration, DR stage, surgical interval, and cumulative phacoemulsification energy. Interaction analysis was performed to explore whether surgical interval modified the association between HbA1c and Δcytokines. A 2-tailed *P* value < .05 was considered statistically significant. Because of the retrospective design, an a priori sample size calculation was not performed. Nevertheless, the final sample size was comparable to or larger than that of previously published aqueous humor cytokine studies in diabetic cataract patients and was sufficient to detect moderate effect sizes in inter-eye cytokine differences.

## 3. Results

### 3.1. Baseline characteristics

A total of 88 patients (cataract surgery was performed on each eye separately) were included in the final analysis. The mean age was 64.3 ± 7.2 years, and 46 (52.3%) were male. The mean duration of diabetes was 12.5 ± 5.4 years, and the mean HbA1c was 8.1 ± 1.2%. According to HbA1c levels, 27 patients (30.7%) were classified into group A (<7.0%), 38 (43.2%) into group B (7.0–8.9%), and 23 (26.1%) into group C (≥9.0%). Patients with higher HbA1c tended to have longer diabetes duration, higher body mass index, and a greater frequency of moderate-to-severe DR (*P* for trend = .08). No significant differences were observed among the 3 groups regarding age, sex distribution, smoking, or alcohol consumption (all *P* > .05; Table 1).

**Table 1:** Baseline characteristics of the study population.

Variable	Group A (<7.0%)	Group B (7.0–8.9%)	Group C (≥9.0%)	*P* value
n (%)	27 (30.7%)	38 (43.2%)	23 (26.1%)	–
Age (yr)	64.1 ± 7.4	63.9 ± 6.9	65.0 ± 7.5	.72
Male, n (%)	13 (48.1%)	20 (52.6%)	13 (56.5%)	.83
BMI (kg/m²)	24.6 ± 2.8	25.4 ± 3.1	26.2 ± 2.9	.09
Duration of diabetes (yr)	11.8 ± 5.1	12.6 ± 5.6	13.4 ± 5.3	.56
Education level, n (%)				
Primary or below	8 (29.6%)	12 (31.6%)	8 (34.8%)	.89
Secondary	14 (51.9%)	19 (50.0%)	10 (43.5%)	
College or above	5 (18.5%)	7 (18.4%)	5 (21.7%)	
Smoking status, n (%)				
Current smoker	6 (22.2%)	9 (23.7%)	7 (30.4%)	.67
Former smoker	8 (29.6%)	10 (26.3%)	6 (26.1%)	
Never smoked	13 (48.2%)	19 (50.0%)	10 (43.5%)	
Alcohol consumption, n (%)				
Current drinker	5 (18.5%)	8 (21.1%)	6 (26.1%)	.79
Nondrinker	22 (81.5%)	30 (78.9%)	17 (73.9%)	
DR stage (mild/moderate/severe)	10/12/5	13/18/7	8/10/5	.78
HbA1c (%)	6.5 ± 0.3	8.0 ± 0.5	9.6 ± 0.4	<.001

BMI = body mass index, DR = diabetic retinopathy, HbA1c = glycated haemoglobin.

### 3.2. Comparison of aqueous cytokine levels between first-eye and second-eye surgeries

Across all diabetic patients, the aqueous humor concentrations of IL-6, IL-8, TNF-α, and MCP-1 were markedly higher during the second-eye surgery than the first-eye surgery (all *P* < .01), whereas IL-10 showed no significant difference (*P* = .27). The median inter-eye increase (Δcytokine) was most evident for IL-6 (2.15 pg/mL, IQR 1.10–3.40) and IL-8 (1.72 pg/mL, IQR 0.90–2.85), both representing moderate-to-large effect sizes (Cohen’s *d* = 0.68 and 0.61, respectively). These results indicate a consistent amplification of intraocular inflammatory activity in the second-eye procedures. Further subgroup analysis demonstrated that the magnitude of cytokine elevation was related to glycemic control. Patients in group C (HbA1c ≥ 9.0%) showed significantly higher ΔIL-6 and ΔIL-8 values compared to those in group A (HbA1c < 7.0%; *P* < .01), while patients in group B (7.0–8.9%) exhibited intermediate changes. This trend persisted even after adjusting for age, sex, diabetes duration, and DR severity (Table 2).

**Table 2:** Comparison of aqueous cytokine levels between first-eye and second-eye surgeries and subgroup analysis by HbA1c.

Cytokine (pg/mL)	First-eye (mean ± SD)	Second-eye (mean ± SD)	Δ (second–first, mean ± SD)	Effect size (Cohen’s *d*)	*P* value	Group A Δ (<7.0%)	Group B Δ (7.0–8.9%)	Group C Δ (≥9.0%)	*P* for trend
IL-6	4.32 ± 2.14	6.47 ± 2.56	2.15 ± 1.35	0.68	<.001	1.56 ± 0.82	2.10 ± 1.05	3.12 ± 1.54	<.001
IL-8	3.86 ± 1.95	5.58 ± 2.42	1.72 ± 1.15	0.61	<.001	1.10 ± 0.73	1.82 ± 0.96	2.34 ± 1.28	.002
TNF-α	2.41 ± 0.96	2.68 ± 1.10	0.27 ± 0.58	0.24	.08	0.21 ± 0.46	0.29 ± 0.54	0.31 ± 0.62	.41
MCP-1	9.62 ± 4.22	11.15 ± 4.76	1.53 ± 1.98	0.30	.06	1.20 ± 1.10	1.56 ± 1.80	2.01 ± 2.20	.09
IL-10	1.75 ± 0.72	1.82 ± 0.74	0.07 ± 0.18	0.10	.27	0.05 ± 0.12	0.08 ± 0.15	0.10 ± 0.20	.33

IL-6 = interleukin-6, IL-8 = interleukin-8, IL-10 = interleukin-10, MCP-1 = monocyte chemoattractant protein-1, SD = standard deviation, TNF-α = tumor necrosis factor-alpha.

### 3.3. Association between HbA1c and inter-eye cytokine differences

Correlation and regression analyses were performed to explore the relationship between systemic glycemic control and inter-eye inflammatory variations. Spearman correlation analysis demonstrated that HbA1c levels were positively correlated with ΔIL-6 (*r* = 0.48, *P* < .001) and ΔIL-8 (*r* = 0.42, *P* = .002), whereas no significant correlation was found for ΔTNF-α or ΔMCP-1 (*P* > .05). When patients were stratified according to HbA1c categories, those with HbA1c ≥ 9.0% exhibited nearly a 2-fold higher increase in ΔIL-6 compared with patients with HbA1c < 7.0% (3.1 ± 1.5 vs 1.6 ± 0.8 pg/mL, *P* < .001). Similarly, ΔIL-8 levels showed a gradual rise across HbA1c tertiles (*P* for trend = .002). Multivariable linear regression analysis confirmed HbA1c as an independent predictor of ΔIL-6 (β = 0.37, 95% confidence interval: 0.19–0.54, *P* < .001) and ΔIL-8 (β = 0.29, 95% confidence interval: 0.10–0.48, *P* = .003), after adjusting for potential confounders including age, sex, diabetes duration, DR stage, surgical interval, and cumulative ultrasound energy. Among the covariates, surgical interval also exhibited a significant positive association with ΔIL-6 (β = 0.12, *P* = .017). The adjusted *R*² values of the final models were 0.38 for ΔIL-6 and 0.32 for ΔIL-8, demonstrating good explanatory power (Table 3).

**Table 3:** Multivariable linear regression analysis of factors associated with ΔIL-6 and ΔIL-8.

Variable	β (ΔIL-6)	95% CI (ΔIL-6)	*t* value (ΔIL-6)	*P* value (ΔIL-6)	β (ΔIL-8)	95% CI (ΔIL-8)	*t* value (ΔIL-8)	*P* value (ΔIL-8)
HbA1c (%)	0.37	0.19–0.54	4.12	<.001	0.29	0.10–0.48	3.08	.003
Surgical interval (d)	0.12	0.02–0.22	2.43	.017	0.09	0.01–0.19	2.01	.048
DR stage	0.09	−0.04 to 0.21	1.55	.12	0.06	−0.06 to 0.18	1.10	.28
Age (yr)	0.04	−0.03 to 0.12	1.02	.31	0.02	−0.05 to 0.10	0.68	.50
Diabetes duration (yr)	0.06	−0.05 to 0.18	1.26	.21	0.05	−0.06 to 0.17	1.04	.30
Adjusted *R*²	0.38	–	–	–	0.32	–	–	–

DR = diabetic retinopathy, HbA1c = glycated hemoglobin, IL-6 = interleukin-6, IL-8 = interleukin-8, IL-10 = interleukin-10.

### 3.4. Influence of surgical interval

Subgroup and interaction analyses were performed to determine whether the timing between sequential cataract surgeries influenced inter-eye inflammatory variations. Patients with shorter surgical intervals (≤14 days) exhibited significantly smaller inter-eye increases in IL-6 and IL-8 compared with those whose surgeries were separated by ≥28 days (*P* < .05). The mean ΔIL-6 rose progressively from 1.32 ± 0.96 pg/mL in the ≤14-day group to 2.88 ± 1.46 pg/mL in the ≥ 28-day group (*P* for trend < .001), while ΔIL-8 increased from 1.10 ± 0.85 to 2.33 ± 1.28 pg/mL (Table 4).

**Table 4:** Inter-eye cytokine differences according to surgical interval.

Surgical interval (d)	n	ΔIL-6 (mean ± SD)	ΔIL-8 (mean ± SD)	Effect size (Cohen’s *d*)	*P* (vs ≤ 14 d)	*P* for trend
≤14	28	1.32 ± 0.96	1.10 ± 0.85	–	–	–
15–27	31	2.09 ± 1.21	1.78 ± 1.10	0.59	.031	.014
≥28	29	2.88 ± 1.46	2.33 ± 1.28	0.97	<.001	<.001

IL-6 = interleukin-6, IL-8 = interleukin-8, SD = standard deviation.

## 4. Discussion

In this retrospective study, we demonstrated that both systemic glycemic control and surgical interval significantly influence intraocular inflammatory responses during sequential cataract surgeries in patients with T2DM. Specifically, IL-6 and IL-8 concentrations were markedly higher during second-eye surgeries, and these inter-eye cytokine differences correlated strongly with HbA1c levels and surgical timing. These findings provide novel evidence that metabolic dysregulation and procedural scheduling act synergistically to modulate ocular immune reactivity.

### 4.1. Hyperglycemia and ocular inflammatory priming

Chronic hyperglycemia has long been recognized as a systemic pro-inflammatory state. Elevated glucose levels lead to increased production of reactive oxygen species, activation of the nuclear factor kappa-light-chain-enhancer of activated B cells pathway, and formation of advanced glycation end products, which together promote endothelial dysfunction and microvascular permeability.^[8,9]^ These mechanisms contribute to a breakdown of the blood–aqueous barrier and facilitate cytokine entry into the anterior chamber.

Our results showing HbA1c as an independent predictor of ΔIL-6 and ΔIL-8 align with earlier reports demonstrating elevated aqueous cytokines in diabetic eyes. Chen et al reported that IL-6 and its soluble receptor were significantly increased in diabetic cataract patients compared with controls.^[1]^ Li et al further demonstrated that IL-6 upregulation correlated with metabolic indices and could be modulated by systemic therapy such as metformin.^[10]^ Meta-analyses have also confirmed that IL-6 and IL-8 are consistently higher in diabetic aqueous or vitreous samples, particularly in eyes with advanced DR.^[11,12]^

These cytokines play essential roles in leukocyte recruitment, vascular leakage, and fibrovascular proliferation.^[13]^ Hence, persistently elevated IL-6 and IL-8 in hyperglycemia may create a primed inflammatory milieu, predisposing diabetic eyes to exaggerated postoperative responses.

### 4.2. Inflammatory amplification in second-eye surgery

The observed rise in inflammatory mediators in second-eye surgeries supports the hypothesis of cross-eye immune sensitization. After the first-eye procedure, surgical trauma releases damage-associated molecular patterns and cytokines into the circulation, which can activate monocytes, macrophages, and endothelial cells systemically.^[14]^ Tang et al reported that unilateral cataract extraction led to detectable cytokine changes in the contralateral eye, suggesting a systemic immune crosstalk effect.^[15]^

Moreover, Dong et al revealed elevated IL-6, IL-8, MCP-1, and vascular endothelial growth factor in aqueous samples from diabetic cataract patients, correlating with subclinical postoperative inflammation.^[16]^ These results collectively indicate that immune activation from the first-eye surgery may not fully resolve before the second procedure, especially in the setting of metabolic stress.

### 4.3. Role of surgical interval

Our subgroup and interaction analyses revealed that surgical interval was a significant modifier of the relationship between HbA1c and ΔIL-6. Patients who underwent second-eye surgery within ≤14 days exhibited the lowest cytokine changes, whereas those with ≥28-day intervals had the highest increases. This suggests a time-dependent immune amplification, likely mediated by persistent activation of systemic inflammatory pathways or delayed recovery of the ocular microenvironment.

Similar time-related immune sensitization phenomena have been reported in experimental models of ocular surgery and trauma.^[17]^ In human studies, elevated aqueous flare and cell counts have been observed when the second-eye surgery is delayed beyond several weeks.^[18]^ Collectively, these findings indicate that the postoperative immune system remains “primed” for several weeks, and prolonged intervals may enhance reactivation upon the next procedure.

### 4.4. Clinical implications

These findings have direct clinical implications for managing diabetic cataract patients. Preoperative optimization of glycemic control remains paramount – our data show that patients with HbA1c ≥ 9.0% experienced nearly a twofold greater ΔIL-6 than those <7.0%, consistent with prior clinical evidence.^[19,20]^ This inflammatory amplification may underlie the higher incidence of postoperative cystoid macular edema, corneal edema, or fibrin reaction observed in diabetics.

Second, the surgical interval should be considered a modifiable risk factor. Shorter intervals (≤14 days) may minimize immune reactivation, particularly in patients with well-controlled glucose. When prolonged intervals are unavoidable, more intensive anti-inflammatory prophylaxis – such as extended corticosteroid or nonsteroidal regimens – may be warranted.^[20]^

In addition, IL-6 and IL-8 could serve as biomarkers for identifying high-risk patients before the second surgery. As point-of-care cytokine assays become more feasible, they might help tailor anti-inflammatory strategies to individual metabolic and immune profiles.

### 4.5. Comparison with prior studies

Previous studies have reported elevated postoperative inflammation in diabetic eyes, but few have linked it quantitatively to HbA1c or surgical interval. Our findings extend prior evidence by demonstrating that both metabolic and procedural factors jointly influence cytokine expression. Dong et al^[11]^ and Funatsu et al^[16]^ reported similar IL-6 and MCP-1 elevations in diabetic aqueous humor. Hamid et al also showed that diabetic eyes display higher inflammatory responses and delayed blood–aqueous barrier recovery after phacoemulsification.^[20]^

The present study adds a temporal and systemic perspective, suggesting that second-eye inflammatory enhancement results from combined effects of metabolic priming, immune memory, and procedural timing. Such understanding provides a foundation for designing individualized surgical schedules and perioperative anti-inflammatory management in diabetic populations.

### 4.6. Limitations

Several limitations warrant consideration. First, the retrospective design may introduce selection bias, and causal relationships cannot be firmly established. Prospective studies with standardized follow-up protocols are needed to validate these findings. Second, the sample size, although sufficient for multivariate analysis, remains modest; larger multicenter cohorts would enhance generalizability. Third, only a limited cytokine panel was analyzed, focusing on IL-6, IL-8, TNF-α, MCP-1, and IL-10. Broader proteomic or transcriptomic profiling could reveal additional pathways linking metabolic stress to ocular inflammation. Although surgical interval was included as a covariate in multivariable analyses, residual confounding cannot be completely excluded. In particular, surgical timing may have been influenced by nonmedical factors such as scheduling constraints or patient preference, which could not be fully quantified in this retrospective study. Given the retrospective design, selection bias cannot be completely excluded. In particular, only patients with successful bilateral aqueous humor sampling and uncomplicated surgeries were included, which may limit the generalizability of the findings to higher-risk diabetic populations.

Although formal multicollinearity testing did not reveal statistically significant collinearity among covariates, metabolic parameters such as HbA1c level and diabetes duration are biologically related. Therefore, subtle interdependencies between these variables may persist and could contribute to residual confounding. Given the retrospective observational design, causal inference cannot be established, and the independent effects of long-term glycemic exposure and current glycemic control may not be completely separable. Prospective studies with longitudinal metabolic profiling are needed to further disentangle these effects.

Finally, postoperative clinical outcomes, such as anterior chamber flare or visual recovery, were not directly correlated with cytokine levels, which should be addressed in future studies.

## 5. Conclusion

In conclusion, elevated HbA1c levels were significantly associated with greater inter-eye increases in IL-6 and IL-8 during sequential cataract surgeries in patients with type 2 diabetes mellitus. These findings suggest that systemic glycemic status and surgical timing are important factors associated with intraocular inflammatory activity. Optimizing systemic glucose control and minimizing the time between surgeries may help reduce postoperative inflammation and improve visual outcomes in diabetic cataract patients.

## Author contributions

**Conceptualization:** Jialing He, Xiaochun Li, Rui Gong.

**Data curation:** Jialing He, Xiaochun Li, Rui Gong.

**Formal analysis:** Jialing He, Xiaochun Li, Rui Gong.

**Funding acquisition:** Xiaochun Li, Rui Gong.

**Investigation:** Xiaochun Li, Rui Gong.

**Methodology:** Rui Gong.

**Writing – original draft:** Jialing He, Xiaochun Li, Rui Gong.

**Writing – review & editing:** Jialing He, Xiaochun Li, Rui Gong.
